# Direct Losses and Media Exposure to Death: The Long-Term Effect of Mourning during the COVID-19 Pandemic

**DOI:** 10.3390/jcm13133911

**Published:** 2024-07-03

**Authors:** Barbara Caci, Giulia Giordano

**Affiliations:** Department of Psychology, Educational Science and Human Movements, University of Palermo, 90128 Palermo, Italy; giulia.giordano@unipa.it

**Keywords:** COVID-19 pandemic, mental health, anxiety, depression, stress, mourning, vicarious grief, media exposure, neuroticism

## Abstract

**Background**: The social distancing policies adopted during the COVID-19 pandemic forced many individuals to confront their mortality and worry about losing loved ones, making it impossible to say goodbye to them properly. Those not directly experiencing loss were inundated with information about COVID-19-related deaths throughout social media, leading to vicarious grief. This study delved into the long-term effects of direct and vicarious mourning on people’s mental health during the COVID-19 pandemic. **Method**: A sample of 171 adults (65% female) aged 19–66 years (Mage = 25.8, SD = 8.57) voluntarily participated in an online survey assessing self-reported psychological measures of complicated grief, stress, depression, dispositional neuroticism, trait anxiety, and situational anxiety. **Results**: MANOVAs revealed that direct mourning experiences had an extremely severe impact on anxiety, stress, and fear of COVID-19, and a moderate effect on those without personal losses. Indeed, participants reporting high media exposure showed higher scores of depression and stress. **Conclusions**: Findings from the current study displayed that during the COVID-19 pandemic, people engaged more in proximal defenses than distal ones, taking health-protective measures, experiencing increased anxiety levels toward virus infection, and feeling distressed. Additionally, vicarious mourning was more strongly associated with depression due to emotional empathy with others.

## 1. Introduction

One of the most long-term effects of the recent COVID-19 pandemic is the changing of death representation, and the subsequent grief elaboration, causing psychological consequences for people’s mental health [1]. The Terror Management Theory (TMT) [2] and the subsequent development of the anxiety buffer disruption theory [3] provide insights into how individuals cope with exposure to death-related stimuli. The central postulate of TMT is that the awareness of their mortality—i.e., the mortality salience effect—leads individuals to perceive prominent levels of stress and anxiety due to the lack of control over the inevitability of death in some circumstances. Such knowledge, indeed, is a human prerogative due to individuals’ cognitive ability and abstract thought, which, contrasting with self-preservation, posits a challenging and harsh living situation, thus enhancing a coping strategy against anxiety. For instance, to cope with death anxiety, individuals postulate their immortality after they die [4]. Empirical studies in the field of TMT have reported that in uncertain situations related to the incapacity to cope with uncontrolled illness, the threat of illness triggers the fear of death [5]. This was the case for the COVID-19 pandemic, which enhanced death anxiety, serving as a stressful life situation [6]. During the COVID-19 pandemic, the diffusion of the SARS-CoV-2 virus infection caused most people to face their mortality and worry about losing their loved ones [7]. People became afraid of getting sick and dying, avoided public spaces and private meetings for risks of infection, and experienced helplessness to protect loved ones, loss, remoteness, detachment, and death increasingly frequently [8]. A new form of situational anxiety, defined as the fear of COVID-19, arose worldwide [9], affecting Israel, Bangladesh, Japan, Brazil, Italy, and the Australian population, also presenting symptoms of depression, suicidal ideation, psychological distress, and increased alcohol consumption [9,10,11,12,13]. These things considered, the COVID-19 pandemic led to people feeling stuck in processing their mourning, having difficulty moving on, and persistent yearning for the mourning. The grief reactions increased because of sudden death, untimely mourning, inadequate care, and social isolation, causing most people to become overwhelmed with grief [14]. Social distancing policies made it impossible to say goodbye to loved ones, and those who encountered the deceased isolated themselves, even though they were experiencing direct mourning. Furthermore, people who did not directly have mourning experienced an overloading of information on all mass media about deaths related to the COVID-19 pandemic. In line with TMT, people enhanced their media exposure to gain information about the spread of the pandemic, and sought control over illness-related worries [15]. However, a sort of infodemic, with contradictory but catastrophic information, defined and presented people with death anxiety, stress, and fear of not controlling the pandemic effects [16,17]. As reported by the existing literature on public mourning in times of disaster or tragedy [16], people during the COVID-19 pandemic not only experienced direct grief related to the mourning of beloved ones, but also the so-called vicarious grief [18], related to the grief experienced in response to someone else’s mourning. People experiencing vicarious grief displayed reactions akin to, yet distinct from, those directly mourning, including weeping, feelings of emptiness and heaviness, sleep and appetite disturbances, and preoccupation. As well, the prevalence rates of acute or complicated grief disorders have risen drastically during the COVID-19 pandemic [19]. Complicated grief disorder (CG) is a mental disorder classified in the DSM-5 as presenting symptoms that emerge after the death of a family member or close friend (i.e., mourning). People with CG experience profound grief and loss, leading to clinically significant distress. They might display symptoms including depression, emotional pain, numbness, loneliness, disturbances in their sense of identity, and challenges in managing interpersonal relationships. Difficulty accepting the loss is also anticipated, and can manifest as ongoing rumination about the death, an intense longing for reunion with the deceased, or disbelief that the death has happened. Recent studies corroborated increasing CG and post-traumatic stress, depression, and anxiety symptoms among American adults bereaved due to COVID-19 [20,21], as well as among Brazilian [22] and Chinese adults [23]. As well, studies showed that the intensity of CG in those bereaved by COVID-19 mourning is much higher in comparison to those bereaved by other causes of death [24]. 

Moreover, the present study focuses on media exposure to death-related information about the COVID-19 pandemic, and psychological distress related to direct and vicarious grief. Consistent with previous research, media exposure offers extensive and detailed information about stressful events, which can heighten awareness of one’s vulnerability and mortality, potentially leading to anxiety and stress-related disorders [25]. Indeed, the constant stream of accurate and fake news incites confusion, uncertainty, and panic, enhancing individuals’ mental health symptoms [26,27,28]. Furthermore, personality traits such as neuroticism, defined under the Five Factors model [29], and fear of COVID-19 could contribute to modulating individuals’ distress levels [30,31]. Thus, the current study first aims to explore the linear associations between CG, distress, neuroticism, and fear of the COVID-19 pandemic in a sample of Italian adults to evaluate the risks of increasing mental health problems in the general population as a long-term effect of the COVID-19 pandemic. Second, it aims to verify the effects of media exposure on CG and distress in people experiencing indirect mourning. 

## 2. Materials and Methods

### 2.1. Participants

The study included a convenience sample of 171 adults (F = 65%) aged 19–66 years (mean age = 25.8; SD ± 8.57; range: 19–66), most of whom were Italian (92.3%), with a high school degree (46.2%), or a first/secondary-level degree (44.5%), voluntary enrolled without compensation. The Declaration of Helsinki was carried out in the study, and we adopted ethical standards for conducting research in the social sciences with humans. All participants provided written informed consent after receiving a complete description of the study’s aim. The Bioethical Committee of the University of Palermo approved all recruitment and assessment procedures (n. 140/2023). 

### 2.2. Measures and Procedure

All participants took part in an online survey, and data were collected by a Google form comprising the following self-report measures:

– COVID-19 Personal sheet: It aimed at registering socio-demographic variables (i.e., gender, age, nationality, occupation, and educational level). To evaluate the impact of the COVID-19 pandemic at an individual level, three questions ask subjects to describe (1) if they were positive for SARS-CoV-2 infection in the last year; (2) the gravity level of their symptoms on a scale from 1–10 points; and (3) their health problems during the COVID-19 pandemic. In addition, one question asked participants to indicate whether a person close to them had contracted the SARS-CoV-2 infection with lethal consequences, specifying the degree of kinship and affinity (i.e., as a 0/1 measure of the personal mourning). If the participants declared that they had not had significant mourning due to the SARS-CoV-2 infection, they were asked another question indicating on a scale from 1–10 how often they believed they had been exposed to media reports dealing with death due to the COVID-19 pandemic (i.e., as a measure of media exposure to mourning).
– Inventory Complicated Grief (ICG–Italian adaptation form) [32]: It is a 19-item self-report questionnaire aimed at measuring complicated grief. Each item scores on a Likert scale with anchors from 0 = never to 3 = always. The total score was obtained by summing up the participants’ scores for each questionnaire item. It comprised scores from 0–74, with scores greater than 25 points indicating high levels of complicated grief. In the current study, the ICG-standardized Cronbach’s alpha value was about 0.972, showing excellent internal consistency.
– Depression Anxiety Stress Scales-21 (DASS-21) [33]: It is a 21-item self-report scale offering a measure of psychological distress in the three different components of depression, anxiety, and stress (each subscale has 7 items). Each item scores on a Likert scale with anchors from 0 = never to 3 = always. To calculate the total scores for each subscale, the score of each item group was multiplied by two [34]. The clinical cut-offs for each scale’s scores were as follows:
  - Depression subscale (Cronbach’s alpha = 0.935): average score: 0–9 points; mild score: 10–13 points; moderate score: 14–20 points; severe score: 21–27 points; extremely severe score: more than 28 points.
  - Anxiety subscale (Cronbach’s alpha = 0.884): average score: 0–7 points; mild score: 8–9 points; moderate score: 10–14 points; severe score: 15–19 points; extremely severe score: more than 20 points.
  - Stress subscale (Cronbach’s alpha = 0.927): average score: 0–14 points; mild score: 15–18 points; moderate score: 19–25 points; severe score: 26–33 points; extremely severe score: more than 34 points.
– Personality Inventory [35]: It is a 20-item self-report questionnaire measuring personality in light of the Big Five theory [29]. It comprises five subscales, each having four items, related to extraversion, conscientiousness, agreeableness, neuroticism, and openness. Each item scores on a Likert scale with anchors from 1 = never to 5 = always. In the current study, we apply only the neuroticism subscale (Cronbach’s alpha = 0.665). The total score was calculated by summing participants’ scores for each item of the scale, and it ranged from 0–20 points, with high scores indicating high levels of neuroticism.
– Fear of COVID-19 scale (FCV-19S) [36]: It is a 7-item self-report questionnaire aimed at measuring a form of situational anxiety related to the fear of being infected by the SARS-CoV-2 virus. Each item scores on a Likert scale with anchors from 1 = strongly disagree to 5 = strongly agree. The total score was computed by summing participants’ scores for each item of the scale, and it comprised 7–35 points, with high scores indicating high levels of fear of COVID-19. In the current study, the FCV-19S-standardized Cronbach’s alpha value was about 0.916, showing excellent internal consistency.

All participants were recruited using a snowballing procedure, posting the form on multiple social media groups of students attending the psychology courses at the University of Palermo (e.g., Facebook, Instagram, WhatsApp groups). The inclusion criteria were as follows: (1) being 18 years old and over, (2) having experienced the personal mourning of someone due to the COVID-19 pandemic, and (3) having been exposed to the mourning of someone due to the COVID-19 pandemic in media (e.g., television, social media). Data were collected in the period between April and June 2023, about a year after the abolition of the sanitary emergency state in Italy (Italian law decree n. 24, 24 March 2022), and the declaration by the World Health Organization of the end of the COVID-19 pandemic (WHO, 5 May 2023). This choice allowed us to evaluate the participants’ long-term risk of complicated grief both in the case of personal mourning due to COVID-19 infection and in the case of mediatic exposure to mourning. Participants filled out the form in about 10–15 min on average. When sending it, data were automatically registered on an EXCEL sheet, downloaded by researchers at the end of the data collection phase. 

### 2.3. Statistical Analyses

All data were analyzed using the SPSS Statistical Package for Social Science (IBM SPSS Statistics—version 27) and descriptive statistics (e.g., frequencies, media, SD); linear Pearson’s correlations, Cronbach’s alpha values, and Multivariate Analyses of Variance (MANOVA) were calculated.

## 3. Results

### 3.1. Descriptive Statistics

Table 1 shows the impact of the COVID-19 pandemic on all participants’ health. Most participants (64%) were positive for SARS-CoV-2; of these, they were primarily female (66%). However, on average, they affirmed that the gravity of their symptoms was low (mean COVID-19 gravity index = 4.53; range 0–10). Furthermore, most participants affirmed that they did not have significant health problems during the COVID-19 pandemic (56%). On the contrary, those who reported health problems affirmed that they had experienced anxiety (16%), mood alteration (5%), depression (2%), insomnia (10%), apathy (5%), and organic problems (6%).

Only 25% of participants had significant personal mourning due to the COVID-19 pandemic; specifically, their loss regarded parents (4%), grandparents (5%), uncle/auntie (4%), friends (6%), and related relatives, such as brother-in-law, son-in-law, daughter-in-law, or cousins (6%). Seventy-five percent of participants had no personal mourning due to the COVID-19 pandemic. However, it affirmed that they had been significantly exposed to mourning during the pandemic, showing a media exposure to mourning mean value of 7.68 points (range 0–10 points). 

Table 2 shows a linear Pearson’s correlation among the measures of complicated grief, psychological distress, fear of the COVID-19 pandemic, and neuroticism for the whole sample of participants.

Linear Pearson’s correlation values show significant intercorrelations among all of the study variables, evidencing a solid consistency of the measurement assessment. In line with the previous literature [37], complicated grief is related to all of the subscales of psychological distress, evidencing that the mourning experience, even if related to media exposure, has a significant impact on the mental health of participants. 

### 3.2. The Effect of Direct Mourning Due to the COVID-19 Pandemic on Psychological Distress, Neuroticism, and Fear of COVID-19

To evaluate the effect of direct mourning during the COVID-19 pandemic, a Multivariate Analysis of Variance (MANOVA) was performed on scores measuring psychological distress, neuroticism, and fear of COVID-19, comparing people who had a personal loss (G1) and people who did not (G2). Results show a significant multivariate effect of the Group on the dependent variables, wherein F(5, 165) = 6.20, *p* < 0.01, η^2^ = 1; furthermore, there are significant univariate effects between the two groups on scores from the DASS-21 Anxiety and the Fear of COVID-19 scales, as reported in Table 3. 

Significant differences were found between the two groups on scores for the DASS-21 Anxiety subscale, with people having direct mourning showing extremely severe scores, whereas people without personal loss showed moderate scores. The results also evidence a significantly similar trend of higher scores for people having direct mourning compared to those without losses, as shown with the Fear of COVID-19 test. No significant differences have emerged in the scores for the DASS-21 Stress and Depression subscale, nor for the PI neuroticism scale. 

### 3.3. The Effect of Complicated Grief during the COVID-19 Pandemic on Psychological Distress, Neuroticism, and Fear of COVID-19 of People with Personal Losses

To evaluate the effect of complicated grief in people experiencing direct mourning during the COVID-19 pandemic, the G1 sample was divided into two subgroups, the High ICG Group, and the Low ICG Group, using the median value of ICG total score equal to 17. Then, a Multivariate Analysis of Variance (MANOVA) on scores at measures of psychological distress, neuroticism, and fear of COVID-19 was performed. Results show a non-significant multivariate effect of the Group on the dependent variables, wherein F(5, 35) = 1.40, *p* = 0.24, η^2^ = 0.43, but significant univariate effects between the two groups on scores for all DASS-21 subscales, as reported in Table 4. 

### 3.4. The Effect of Media Exposure of Mourning during the COVID-19 Pandemic on Psychological Distress, Neuroticism, and Fear of COVID-19 in People without Personal Losses

To analyze the effect of the media exposure to the mourning of unrelated people on psychological distress, a MANOVA was performed on the studied variables on data related to G2 (i.e., people who did not have a personal loss due to the COVID-19 pandemic). Specifically, using the median value on the media exposure indicator, equal to eight, the G2 sample was divided into High and Low Exposure Groups. Then, a MANOVA was performed on independent groups to measure scores for all of the psychological measures. Results show a significant multivariate effect of the Group on the dependent variables, wherein F(5, 124) = 3.3, *p* < 0.01, η^2^ = 0.89, and significant univariate effects between the two groups on scores for DASS-21 Depression and Stress subscales, with HEG having higher scores than LEG, as reported in Table 5. No significant difference has emerged between the two subgroups on the DASS-21 Anxiety subscale, Fear of COVID-19 questionnaire, or PI neuroticism scale. 

## 4. Discussion

The present study aimed to analyze the long-term impact of the COVID-19 pandemic on people’s psychological distress and dispositional or situational anxiety due to personal mourning of loved ones who contracted the SARS-CoV-2 infection with lethal consequences, as well as the media exposure to the mourning of not-related people. 

Pearson’s correlational test results evidenced a significant correlation between all of the studied variables that is in line with the prior literature, showing a general effect of social distancing measures and social isolation on mental health during the COVID-19 pandemic in Italy [38,39]. As well, results comparing people who had direct mourning because of the COVID-19 pandemic (i.e., G1) and those who did not (i.e., G2) show that G1 is more anxious and stressed, and presents higher levels of fear of being infected by SARS-CoV-2. This result is obviously due to the pandemic experience of death. Nevertheless, even if death is a natural part of human life, the COVID-19 pandemic presented novel and unexpected characteristics people have never experienced before. During the COVID-19 pandemic, death usually occurred during hasty medical crises, in the condition of patients’ isolation, loneliness, and alienation, bringing a sort of dehumanization of bereavement; thus, people who lost their relatives experienced the death of their family members and the subsequent separation from them as being more traumatic than usual [40]. The isolation due to the social distancing measures for preventing the spread of the COVID-19 pandemic, the lack of physical contact and adequate emotional support, as well as preparation for death, the suspension of funeral rituals and practices, and the actual loss of family members or relatives can be considered significant stressors during their direct mourning process [41]. Furthermore, in line with prior studies carried out before the COVID-19 pandemic on bereaved families unable to say goodbye to the deceased before death, and those who experienced an excessive sense of guilt [42,43] and a lack of support from their social network [44], a recent study conducted during the COVID-19 pandemic highlighted that unresolved mourning conditions are associated with mental health problems in the examined population [45]. 

The present study also offers a picture of the long-term effect of the death experience during the COVID-19 pandemic, analyzing the mental health consequences both for people with direct mourning and those with vicarious mourning. To this point, the results of the study offer two different pictures, showing that, for G1, comparing people with high vs. low scores of ICG, they endure symptoms of psychological distress and situational anxiety, highlighting a sort of complicated grief. This result aligns with the theoretical framework of TMT [46,47], and highlights the well-known distinction between proximal defenses, which address the problem of death directly, and distal defenses, which lack a logical connection to death but enable individuals to perceive it as a significant, meaningful, lasting, and valuable contribution to the universe [48]. When thoughts of death become conscious, proximal defenses are activated. On one hand, this is done to suppress thoughts and relegate death to a distant future, removing vulnerability to anything that could lead to it; on the other hand, it is done to continue to adopt healthier behaviors to ensure a long life. However, when thoughts of death are on the fringes of consciousness, people focus on faith, mobilizing their distal defenses and considering their cultural vision. Fear is the primary emotion selected by human evolution to survive and, at the same time, defend itself. It also pushes people to adopt protective behaviors to prevent and avoid death in the face of potential danger. The TMT assumes that when thoughts of death are frequent, subjects seek to eliminate them from their consciousness by a simple process of suppression, denial of threat, or taking measures to reduce their vulnerability [4,46]. In line with prior studies [6,49], our results corroborated the idea that people who directly perceived the death of loved ones applied proximal defenses more than distant ones, taking measures to protect their health, such as social distancing, increasing hygiene practices such as hand and surface washing, and the use of protective equipment in public places after the end of the COVID-19 pandemic emergency [50]. However, this is not without personal mental health consequences, since they feel more distressed, thus enhancing their anxiety levels toward the SARS-CoV-2 infection. Under theoretical models [42,51,52], when individuals struggle to cope with existential fears and construct a meaningful life effectively, heightened death anxiety and maladaptive ways of addressing this anxiety are anticipated outcomes. Empirical research has further indicated that reminders of mortality can intensify phobias, compulsive behaviors, depressive symptoms, and anxiety [53]. This phenomenon may help elucidate findings from recent reviews linking pandemics to increased reports of anxiety, depression, and stress [54]. Both death anxiety and ineffective or dysfunctional anxiety management strategies have been identified as potential diagnostic vulnerability factors for mental disorders [51].

The COVID-19 pandemic has brought about an unprecedented experience of vicarious grief, where individuals mourn losses that they have not directly experienced, but have observed in others. This collective grief has been intensified by constant media coverage of the pandemic’s toll, leading to widespread feelings of sorrow and empathy for those affected. Indeed, the results of the current study evidenced significant differences between the HEG and LEG of G2. Specifically, HEG obtained higher LEG scores on the DASS-21 Depression scale, aligning with prior studies. The recent psychological literature examining depression in the context of COVID-19 through TMT posits that the pervasive awareness of death due to the pandemic has exacerbated symptoms of depression, particularly among individuals with pre-existing mental health conditions. For instance, a study found that increased mortality salience during COVID-19 led to heightened depressive symptoms, especially in those with lower levels of self-esteem and social support [52]. Additionally, research suggests that the fear of COVID-19 has amplified existential dread, contributing to a rise in depressive disorders as individuals struggle to find meaning and security in an uncertain world [42]. Likewise, vicarious grief has become a significant psychological phenomenon during the pandemic, as individuals are exposed to stories of loss and suffering on a global scale [24]. The pervasive nature of these narratives, combined with social isolation and uncertainty, has heightened emotional distress even among those who have not personally lost someone to the virus. Similarly, a study highlights that healthcare workers have experienced profound vicarious grief, as they witness ongoing loss and trauma within their professional roles. This form of grief has contributed to increased levels of burnout and mental health challenges among frontline workers [24]. During the COVID-19 pandemic, vicarious grief was observed among health professionals, who reported significant emotional responses like those experienced by direct mourners. This included feelings of anxiety, depressive mood, exhaustion, and tears during grief-related tasks, reflecting common reactions seen in individuals bereaved by COVID-19 deaths.

The stress induced by the COVID-19 lockdown might have triggered relapses in individuals with pre-existing psychiatric conditions, such as depression, anxiety, and psychotic disorders like schizophrenia. The lockdown experienced during the pandemic led to social isolation, which in turn led to people experiencing sensory deprivation and an overall sense of fear and suspicion. As a consequence, this situation might have rein-forced delusional ideas in those people vulnerable to psychiatric disorders [53]. A history of psychiatric problems, as well as inadequate social support, and experiencing a sudden or traumatic death, thus being unprepared for the death, might represent risk factors that can lead to poor bereavement outcomes [54].

The current study’s findings should be considered based on several strengths and limitations. A first strength is that studying the long-term effects of the COVID-19 pandemic provides the significant benefit of enabling researchers and policymakers to understand the enduring psychological impacts, such as chronic anxiety, depression, and PTSD, which may persist long after the immediate crisis has ended. This understanding is crucial for developing effective mental health interventions and support systems tailored to address these lasting issues. A second strength relies on the comparison provided by the current study between both direct and vicarious mourning during the pandemic, enhancing the clinical understanding of the broad spectrum of emotional responses to loss related to psychological distress, recognizing that grief is not limited to personal bereavement, but also includes the empathy and sorrow felt for others. This comprehensive perspective is crucial for developing holistic mental health support systems that address the needs of individuals experiencing direct loss, as well as those affected by the collective trauma of the pandemic. Additionally, it informs the creation of targeted interventions and resources to support mental well-being, fostering resilience in communities facing ongoing and future crises. A final strength is that investigating the long-term mental health effects of the pandemic can inform future crisis management strategies. Insights gained from these studies can guide preparations for potential future public health emergencies, ensuring that mental health considerations are integrated into response plans to protect and support populations better.

Nevertheless, future research is needed to address the limitations of this study. The first limitation relies on the cross-sectional approach and the lack of testing causal associations between variables. Secondly, the study utilized an online questionnaire-based survey, and all variables were assessed through self-report measures, which are susceptible to various biases, such as social desirability bias, as well as inaccuracies due to memory errors, or subjective interpretations of the questions. The current online survey could have limited the sample’s representativeness to people having access to the internet and familiarity with digital tools, potentially excluding specific populations from the study. This could have led to a convenience sample that is not fully representative of the broader population, thus limiting the generalizability of the current findings. The convenience sampling is a starting point, and populations could vary over time and between locations, so further studies should add a probability sampling method. Furthermore, while self-report surveys are convenient and cost-effective, they often lack the depth and nuance that can be obtained through other methods, such as in-depth interviews or observational studies. These alternative methods can provide more prosperous and detailed data that can more effectively capture the complexities of the participants’ experiences. To address these limitations, future research should consider incorporating a mix of methodologies, such as combining self-report surveys with objective measures and qualitative methods. Additionally, efforts should be made to ensure a more diverse and representative sample, potentially through targeted recruitment strategies and providing multiple ways for participants to engage with the study. A final limitation of the current study is that it focuses on the general population, and cannot identify vulnerable populations who are disproportionately affected by the pandemic, such as healthcare workers, individuals with pre-existing mental health conditions, and those experiencing prolonged social isolation. For instance, the mediator role of dispositional traits related to anxiety between social isolation and exposure to death is a topic that warrants further investigation. This area of study holds the potential to deepen our understanding of how dispositional traits influence individuals’ responses to social isolation and exposure to death, making it a compelling avenue for future research. Future studies should be performed to recognize these at-risk groups, as well as to target support and resources to mitigate adverse outcomes.

## 5. Conclusions

In summary, the current study aimed to analyze the long-term effect of the COVID-19 pandemic on mental health, considering psychological distress both in direct mourning and in vicarious mourning. The findings support the idea that mental health consequences for people who had direct losses of beloved ones are related to increasing anxiety levels toward situational stressors (e.g., the fear of being infected by viruses), whereas for people who experienced vicarious mourning through death media exposure, psychological distress is more related to depression. Thus, the study underscores the importance of addressing existential fears and enhancing psychological resilience to mitigate the long-term impact of the pandemic on mental health. Interventions that foster a sense of meaning and connectedness may be particularly effective in reducing psychological distress, such as anxiety and depression linked to heightened mortality awareness during global crises. These findings also emphasize the need for mental health support systems to address vicarious grief, providing clinical treatment aimed at enhancing coping strategies and emotional support to mitigate its impact. Understanding and acknowledging vicarious grief can help clinicians develop targeted interventions to support the general population after the pandemic.

## Figures and Tables

**Table 1:** Positivity to SARS-CoV-2 and health problems in the whole sample (N = 171).

	N (%)					
	N = 171		Males (*n* = 60)		Females (*n* = 111)	
	Yes	No	Yes	No	Yes	No
Positivity to SARS-CoV-2	109 (64%)	62 (36%)	35 (58%)	25 (42%)	75 (66%)	38 (34%)
Health problems	75 (44%)	98 (56%)	20 (30%)	40 (70%)	57 (50%)	56 (50%)

**Table 2:** Pearson’s linear correlations for all of the studied variables (N = 177).

	1	2	3	4	5	6
ICG	-					
DASS-21 Depression	0.391 *	-				
DASS-21 Anxiety	0.560 *	0.800 *	-			
DASS-21 Stress	0.418 *	0.893 *	0.826 *	-		
FCV-19S	0.384 *	0.421 *	0.481 *	0.435 *	-	
PI Neuroticism	0.273 *	0.502 *	0.462 *	0.470 *	0.242 *	-

Note—* Correlation is significant at *p* < 0.001 (two tiles); ICG = Inventory Complicated Grief; DASS = Depression Anxiety Stress Scales; FCV-19S = Fear of COVID-19 scale; PI = Personality Inventory.

**Table 3:** Univariate MANOVA results for G1 and G2.

	G1—Direct Mourning (*n* = 41; F = 28; M = 13)		G2—No Mourning (*n* = 130; F = 84; M = 46)		F (5, 165)	*p*	η^2^
	M	DS	M	DS			
DASS-21 Depression	23.02	14.8	20.8	17.4	0.50	0.50	0.10
DASS-21 Anxiety	20.6	13.2	14.09	14.1	6.9	<0.001	0.74
DASS-21 Stress	29.02	14.05	24.9	17.3	1.8	0.17	0.27
FCV-19S	18.6	7.4	13.1	6.2	21.7	<0.001	0.99
PI Neuroticism	11.8	3.8	11.1	3.3	1.1	0.28	0.18

Note—DASS = Depression Anxiety Stress Scales; FCV-19S = Fear of COVID-19 scale; PI = Personality Inventory.

**Table 4:** Univariate MANOVA results for the High vs. Low ICG Groups. High ICG Group in G1 (i.e., people with direct mourning).

	High ICG Group (*n* = 20; F = 14; M = 6)		Low ICG Group (*n* = 21; F = 14; M = 7)		F (5, 35)	*p*	η^2^
	M	DS	M	DS			
DASS-21 Depression	28.3	12.9	18.0	15.0	5.4	<0.05	0.62
DASS-21 Anxiety	26.1	12.4	15.5	12.0	7.6	<0.01	0.76
DASS-21 Stress	34.1	12.9	24.1	14.6	5.2	<0.05	0.60
FCV-19S	20.3	7.0	17.1	7.6	1.9	0.17	0.27
PI Neuroticism	12.7	3.7	11.0	3.7	2.2	0.14	0.30

Note—DASS = Depression Anxiety Stress Scales; FCV-19S = Fear of COVID-19 scale; PI = Personality Inventory.

**Table 5:** Univariate MANOVA results for the High vs. Low Exposure Groups. High Exposure Group in G2 (i.e., people with vicarious mourning).

	High Exposure Group (*n* = 56; F = 40; M = 16)		Low Exposure Group (*n* = 74; F = 44; M = 30)		F	*p*	η^2^
	M	DS	M	DS			
DASS-21 Depression	26.0	19.2	16.1	14.4	11.2	<0.001	0.91
DASS-21 Anxiety	16.2	15.5	12.4	12.6	2.3	0.12	0.33
DASS-21 Stress	29.0	17.8	21.3	15.6	6.9	<0.01	0.74
FCV-19S	14.2	6.5	12.7	6.2	1.7	0.19	0.25
PI Neuroticism	11.4	3.8	11.0	3.2	0.38	0.53	0.09

Note—DASS = Depression Anxiety Stress Scales; FCV-19S = Fear of COVID-19 scale; PI = Personality Inventory.

## Data Availability

The data presented in this study are available on request from the corresponding author due to the approval of the Bioethics Committee of the University of Palermo.
